# Direct interaction network and differential network inference from compositional data via lasso penalized D-trace loss

**DOI:** 10.1371/journal.pone.0207731

**Published:** 2019-07-24

**Authors:** Shun He, Minghua Deng

**Affiliations:** 1 School of Mathematical Sciences, Peking University, Beijing, 10087, P.R. China; 2 Center for Statistical Science, Peking University, Beijing, 10087, P.R. China; Kyushu Institute of Technology, JAPAN

## Abstract

The development of high-throughput sequencing technologies for 16S rRNA gene profiling provides higher quality compositional data for microbe communities. Inferring the direct interaction network under a specific condition and understanding how the network structure changes between two different environmental or genetic conditions are two important topics in biological studies. However, the compositional nature and high dimensionality of the data are challenging in the context of network and differential network recovery. To address this problem in the present paper, we proposed two new loss functions to incorporate the data transformations developed for compositional data analysis into D-trace loss for network and differential network estimation, respectively. The sparse matrix estimators are defined as the minimizer of the corresponding lasso penalized loss. Our method is characterized by its straightforward application based on the ADMM algorithm for numerical solution. Simulations show that the proposed method outperforms other state-of-the-art methods in network and differential network inference under different scenarios. Finally, as an illustration, our method is applied to a mouse skin microbiome data.

## 1 Introduction

Microbes play critical roles in Earth’s biogeochemical cycles [1] and impact the health of humans significantly [2]. Understanding interactions among microbes under a specific condition is a key research topic in microbial ecology [3]. Bandyopadhyay *et al*. [4] also showed that these interactions can change under various environmental or genetic conditions. With the development of high-throughout sequencing technology, 16s rRNA gene sequences can be amplified, sequenced, and grouped into common Operational Taxonomic Units (OTUs), and as a result, microbial abundance information can be obtained for further exploration [5]. One of the major challenges is to discover associations among microbes and how these associations change under different conditions, which could in turn help us to unravel the underlying interaction network and offer an insight into community-wide dynamics.

Correlation analysis is commonly used to infer the interaction network for absolute abundance data. However, applying traditional correlation analysis to compositional data, as only representative of relative abundances of microbial species, may yield spurious results [6, 7]. Recent methods, such as SparCC [7], CCREPE [8, 9], REBACCA [10] and CCLasso [11], have been proposed to address compositional bias and infer the correlation network of microbe communities. However, pairwise correlations contain both direct and indirect interactions, and correlations may arise when microbes are connected indirectly [12]. Thus, the conditional dependence network describing direct interactions is often more intrinsic and fundamental [13, 14].

For absolute abundance, conditional dependence networks are frequently modeled as Gaussian graphical models where direct interactions are correspond to the support of precision matrix [15, 16]. Meinshausen and Bühlmann [17] proposed a neighborhood selection approach to recover the precision matrix row-by-row by fitting a lasso penalized least square regression model [18]. Yuan and Lin [19] derived the likelihood for Gaussian graphical models and suggested using the maxdet algorithm to compute the corresponding lasso penalized estimator. Friedman *et al*. [20] developed a more efficient algorithm called the graphical lasso. Zhang and Zou [21] proposed a new loss function called D-trace loss and introduced a sparse precision matrix estimator as the minimizer of lasso penalized D-trace loss. Several methods have been proposed to infer the direct interaction network from compositional data. Biswas *et al*. [22] suggested learning the direct interactions from compositional data with a Poisson-multivariate normal hierarchical model called MInt. Kurtz *et al*. [12] proposed a method called SPIEC-EASI, which combines centered log-ratio (clr) transformation [6, 23] for compositional data with the neighborhood selection approach [17] or graphical lasso [20] to estimate the precision matrix. Similar to the idea of Yuan and Lin [19], Fang *et al*. [14] first derived likelihood with compositional data for Gaussian graphical models and then estimated the precision matrix with a lasso penalized maximum likelihood method called gCoda. Yuan *et al*. [24] introduced a compositional D-trace loss (CD-trace) based on D-trace loss to estimate the precision matrix. In this paper, we proposed a new loss function called CDTr, with more concise form than CD-trace, to incorporate clr transformation [6, 23] into D-trace loss [21] to estimate the precision matrix from compositional data.

Biological networks often vary according to different environmental or genetic conditions [4]. Understanding how networks change and estimating differential networks are important tasks in biological studies. In recent years, researchers have actively sought methods of estimating differential networks for absolute abundance data. Chiquet *et al*. [25], Guo *et al*. [26] and Danaher *et al*. [27] estimated the precision matrices and their differences jointly by penalizing the joint log-likelihood with different penalties. Zhao *et al*. [28] developed a *ℓ*_1_-minimization method for direct estimation of differential networks, which does not require sparsity of precision matrices or their separate estimation. Yuan *et al*. [29] proposed a new loss function called DTL based on D-trace loss [21] to estimate the precision matrix difference directly. In this paper, we also extended our method to incorporate clr transformation [6, 23] into DTL [29] to estimate the differential network from compositional data.

The remainder of the paper is organized as follows. In Section 2, we introduce our new loss functions to incorporate clr transformations for compositional data analysis into D-trace loss, thereby enabling us to estimate both direct interaction network and differential direct interaction networks from compositional data, respectively. In Section 3, the performance of our method was evaluated and compared with other state-of-the-art methods under various simulation scenarios. In Section 4, the proposed methods are illustrated with an application to a mouse skin microbiome data.

## 2 Materials and methods

### 2.1 Compositional data and clr transformation

We begin with some notations and definitions for convenience. For a *p* × *p* matrix $X=\left(X_{ij}\right)\in R^{p\times p}$, its transposition, trace and determinant are denoted as *X*^T^, *tr*(*X*) and det *X*, respectively. Let ${\left\|X\right\|}_{F}={\left(\sum_{i,j}X_{ij}^{2}\right)}^{1/2}$, ‖*X*‖_∞_ = max_*i*_ ∑_*j*_ |*X*_*ij*_|, ‖*X*‖_1_ = max_*j*_ ∑_*i*_|*X*_*ij*_|, |*X*|_1_ = ∑_*i*,*j*_ |*X*_*ij*_|, and |*X*|_1,off_ = ∑_*i*≠*j*_ |*X*_*ij*_| be the Frobenius norm, ∞-norm, 1-norm, *ℓ*_1_-norm and off-diagonal *ℓ*_1_-norm of *X*. Denote by *vec*(*X*) the *p*^2^-vector from stacking the columns of *X*, and *X* ≻ 0 means that *X* is positive definite. For two matrices $X,Y\in R^{p\times p}$, let *X* ⊗ *Y* be the Kronecker product of *X* and *Y*. We use 〈*X*, *Y*〉 to denote *tr*(*XY*^T^) throughout this paper.

Suppose that there are *p* microbe species and that their absolute abundances are ***z*** = (*z*_1_, *z*_2_, …, *z*_*p*_) respectively. However, instead of absolute abundances, it is often the case that only the relative abundances (or closed compositions) ***x*** = (*x*_1_, *x*_2_, …, *x*_*p*_), where

$$\begin{array}{c} x_{j}=\frac{z_{j}}{\sum_{k=1}^{p}z_{k}},\;j=1,2,\ldots ,p, \end{array}$$ (1)

can be observed in real experiments. If the log-transformed absolute abundances ln ***z*** follow a multivariate Gaussian distribution with mean ***μ*** and nonsingular covariance matrix Σ, the precision matrix Θ = Σ^−1^ depicts the direct interaction network among microbial species since ln *z*_*i*_ and ln *z*_*j*_ are conditionally independent given other components of ***z*** if and only if Θ_*ij*_ = 0 [13]. Moreover, we can describe this direct interaction network with an undirected graph if we represent the *p* microbe species with *p* vertices and connect the conditionally dependent species pairs.

Log-ratios $\text{ln}(\frac{x_{i}}{x_{j}})$ [6, 23] are commonly used in compositional data analysis, since ratios are preserved when the absolute abundances are expressed as relative abundances [12]. Aitchison [6, 23] also proposed a statistically equivalent centered log-ratio (clr) transformation. The centering matrix is $G=I-\frac{1}{p}1_{p}1_{p}^{\text{T}}$, where **1**_*p*_ is a *p*-dimensional all-ones vector and *I* is identity matrix. Applying the clr transformation and using ln ***x*** = ln ***z*** − **1**_*p*_ ln *s* and *G***1**_*p*_ = **0**_*p*_, it follows that

$$\begin{array}{c} G\;\text{ln}\;x=G\;\text{ln}\;z. \end{array}$$ (2)

Denoted by Σ_ln ***x***_ the covariance matrix of the log-transformed relative abundances, we have

$$\begin{array}{c} G\Sigma_{\text{ln}\;x}G=G\Sigma G. \end{array}$$ (3)

Similarly, Eqs (2) and (3) establish a bridge between the observed relative abundances and the unobserved absolute abundances. SPIEC-EASI [12] assumes that *G*Σ_ln ***x***_*G* serves as a good approximation of Σ since *G* − *I* ≈ 0 when *p* ≫ 0, and apply the neighborhood selection approach [17] or graphical lasso [20] to the clr-transformed relative abundances for precision matrix estimation.

### 2.2 CDTr: Compositional network analysis with D-trace loss

From the empirical loss minimization perspective, SPIEC-EASI is not the most natural and concise because of the approximation and the log-determinant term in graphical lasso [20]. In this section, we introduce an innovative loss function to estimate the direct interaction network from compositional data with D-trace loss. The new D-trace loss for compositional data (CDTr loss) is proposed as

$$\begin{array}{c} L_{CD}\left(\Theta ;\Sigma \right)=\frac{1}{2}\left\langle \Theta^{2},G\Sigma G\right\rangle -\left\langle \Theta ,G\right\rangle =\frac{1}{2}\left\langle \Theta^{2},G\Sigma_{\text{ln}\;x}G\right\rangle -\left\langle \Theta ,G\right\rangle . \end{array}$$ (4)

We can view the CDTr loss as an analogue of the D-trace [21] loss $L_{D}\left(\Theta ;\Sigma \right)=\frac{1}{2}\langle \Theta^{2},\Sigma \rangle -\langle \Theta ,I\rangle$. The meaning of incorporating clr transformation into the original D-trace loss is to avoid the unobserved absolute abundance and account for the compositionality. If we know the absolute abundance data, we can simply substitute the finite sample estimator of Σ (denoted by $\hat{\Sigma }$) into D-trace loss and estimate the precision matrix Θ with the corresponding lasso penalized estimator. However, for relative abundances or compositional data, only the finite sample estimator of Σ_ln ***x***_ (denoted by ${\hat{\Sigma }}_{\text{ln}\;x}$) is available, instead of the finite sample estimator of Σ. Thanks to the clr transformation and the bridge Eq (3), we can estimate *G*Σ*G* with $G{\hat{\Sigma }}_{\text{ln}\;x}G$, even though $\hat{\Sigma }$ is not available.

It is easy to check that CDTr loss can be written as

$$\begin{array}{c} L_{CD}\left(\Theta ;\Sigma \right)=\frac{1}{2}{\left\|\Sigma^{1/2}G\Theta -\Sigma^{-1/2}G\right\|}_{F}^{2}-\frac{1}{2}\left\langle \Sigma^{-1},G\right\rangle . \end{array}$$ (5)

To ensure that Σ^−1^ minimizes *L*_*CD*_, namely Σ^1/2^*G*Θ − Σ^−1/2^*G* = 0 when Θ = Σ^−1^, we need the following exchangeable condition:

$$\begin{array}{c} G\Theta =\Theta G\Leftrightarrow G\Sigma =\Sigma G\Leftrightarrow 1_{D}1_{D}^{T}\Sigma =\Sigma 1_{D}1_{D}^{T}. \end{array}$$ (6)

Denote by *σ*_*ij*_ and *ρ*_*ij*_ the covariance and correlation between ln *z*_*i*_ and ln *z*_*j*_, respectively. Then, the exchangeable condition is equivalent to ∑_*l*_ *σ*_*il*_ = ∑_*l*_ *σ*_*jl*_ for all *i*, *j* = 1, 2, …, *p*, which is similar to the assumption ∑_*l*≠*i*_ *σ*_*il*_ = 0, *i* = 1, 2, …, *p* in SparCC [7]. If the variances *σ*_*ii*_, *i* = 1, 2, …, *p* are all the same, then the exchangeable condition simplifies to ∑_*l*≠*i*_ *ρ*_*il*_, *i* = 1, 2, …, *p* are all the same, which implies that the average correlation with other species is nearly the same for each specie. Analogously, the assumption in SparCC simplifies to ∑_*l*≠*i*_ *ρ*_*il*_ = 0, *i* = 1, 2, …, *p*, which implies that the average correlations are very small. In the numerical experiments of section 3, we show that CDTr still performs well, even when the exchangeable condition does not hold.

In practical applications, we use the empirical version of CDTr loss as

$$\begin{array}{c} L_{CD}\left(\Theta ;{\hat{\Sigma }}_{\text{ln}\;x}\right)=\frac{1}{2}\left\langle \Theta^{2},G{\hat{\Sigma }}_{\text{ln}\;x}G\right\rangle -\left\langle \Theta ,G\right\rangle . \end{array}$$ (7)

Since most species do not interact directly when the number of species *p* is large, we further assume that the direct interaction network, or Θ, is sparse, which also helps to solve the under-determined problem caused by compositionality and dimensionality [11, 14, 19]. We employ the commonly used *ℓ*_1_ penalty [18, 19, 21] to handle the sparse assumption, and our sparse estimator of the precision matrix Θ is proposed as

$$\begin{array}{c} {\hat{\Theta }}_{\text{CDTr}}=\underset{\Theta ≻0,\Theta =\Theta^{T}}{\text{argmin}}\frac{1}{2}\left\langle \Theta^{2},G{\hat{\Sigma }}_{\text{ln}\;x}G\right\rangle -\left\langle \Theta ,G\right\rangle +\lambda {\left|\Theta \right|}_{1,\text{off}}, \end{array}$$ (8)

where λ ≥ 0 is the tuning parameter for the tradeoff between the model fitting and the sparsity of ${\hat{\Theta }}_{D}$. Following the idea of Zhao *et al*. [28], the tuning parameter is selected by minimizing the Bayesian Information Criterion (BIC) [30] as

$$\begin{array}{c} \text{BIC}=n\|\left(G{\hat{\Sigma }}_{\text{ln}\;x}G\Theta +\Theta G{\hat{\Sigma }}_{\text{ln}\;x}G\right){/2-G\|}_{1}+\text{log}\left(n\right){\left|\Theta \right|}_{0}, \end{array}$$ (9)

where |Θ|_0_ is the number of non-zero elements in the upper-triangle of Θ, and *n* is the sample size.

Zhang and Zou [21] developed an efficient algorithm based on alternating direction methods [31] for the solution of penalized D-trace loss estimator. We can simply replace $\hat{\Sigma }$ and *I* in D-trace loss with $G{\hat{\Sigma }}_{\text{ln}\;x}G$ and *G* in our CDTr loss and use the algorithm of Zhang and Zou [21] for the numerical solution of (8). Following the idea of Zhang and Zhou [21] and Scheinberg *et al*. [31], we introduce two new matrices, Θ_0_ and Θ_1_. The augmented Lagrangian function of (8) are considered, and Λ_0_, Λ_1_, *ρ* are Lagrangian multipliers. The steps of the ADMM algorithm for the lasso penalized CDTr loss estimator are summaried as follows.

(a) Initialization: k = 0, $\Theta_{0}^{0},\Theta_{1}^{0},\Lambda_{0}^{0}$ and $\Lambda_{1}^{0}$;
(b) $\Theta^{k+1}=H\left(G{\hat{\Sigma }}_{\text{ln}\;x}G+2\rho G,G+\rho \Theta_{0}^{k}+\rho \Theta_{1}^{k}-\Lambda_{0}^{k}-\Lambda_{1}^{k}\right)$;
(c) $\Theta_{0}^{k+1}=S\left(\Theta^{k+1}+\Lambda_{0}^{k}/\rho ,\lambda /\rho \right)$ and $\Theta_{1}^{k+1}={\left[\Theta^{k+1}+\Lambda_{1}^{k}/\rho \right]}_{+}$;
(d) $\Lambda_{0}^{k+1}=\Lambda_{0}^{k}+\rho \left(\Theta^{k+1}-\Theta_{0}^{k+1}\right)$ and $\Lambda_{1}^{k+1}=\Lambda_{1}^{k}+\rho \left(\Theta^{k+1}-\Theta_{1}^{k+1}\right)$;
(e) k = k+1;
(f) Repeat (b)-(e) until convergence.

The definitions of matrix operators *H*(*X*), *S*(*X*) and [*X*]_+_ are listed in S1 Appendix. Compared with CD-trace loss [24] which is also based on D-trace loss and has three terms, our CDTr is more concise with only two terms. The simpler structure of CDTr makes the application of ADMM algorithm straightforward, while a symmetrization step and more auxiliary matrices are needed before applying ADMM algorithm in CD-trace.

### 2.3 DCDTr: Differential compositional network analysis with D-trace loss

Consider that the absolute abundances of *p* microbe species become $z^{*}=\left(z_{1}^{*},z_{2}^{*},\ldots ,z_{p}^{*}\right)$ under another condition and that the relative abundances are $x^{*}=\left(x_{1}^{*},x_{2}^{*},\ldots ,x_{p}^{*}\right)$, respectively. Similarly, we assume $\text{ln}\;z^{*}∼N\left(\mu^{*},\Sigma^{*}\right)$. Thus, we want to estimate the difference between direct interaction networks under different conditions, i.e., the resultant differential network Δ = Σ*^−1^ − Σ^−1^.

A straightforward approach to estimate Δ is to estimate Σ^−1^ and Σ*^−1^ separately and then subtract the estimates under the key assumption that both precision matrices are sparse. However, a more reasonable assumption is that the difference between the precision matrices are sparse, not that both matrices are sparse, since direct interactions may not be sparse while the changes under different conditions are often sparse [29]. Therefore, we proposed a new loss function for differential network estimation with compositional data (DCDTr loss) to estimate Δ directly, under the assumption that the differential network Δ is sparse. The DCDTr loss is proposed as

$$\begin{array}{c} \begin{array}{cc} & L_{DCDTr}\left(\Delta ;\Sigma ,\Sigma^{*}\right)=\frac{1}{4}\left(\left\langle G\Sigma G\Delta ,\Delta G\Sigma^{*}G\right\rangle +\left\langle G\Sigma^{*}G\Delta ,\Delta G\Sigma G\right\rangle \right)+\left\langle \Delta ,G\left(\Sigma^{*}-\Sigma \right)G\right\rangle \\ & =\frac{1}{4}\left(\left\langle G\Sigma_{\text{ln}\;x}G\Delta ,\Delta G\Sigma_{\text{ln}\;x^{*}}G\right\rangle +\left\langle G\Sigma_{\text{ln}\;x^{*}}G\Delta ,\Delta G\Sigma_{\text{ln}\;x}G\right\rangle \right)+\left\langle \Delta ,G\left(\Sigma_{\text{ln}\;x^{*}}-\Sigma_{\text{ln}\;x}\right)G\right\rangle . \end{array} \end{array}$$ (10)

Similarly, our DCDTr loss can be regarded as an analogue to the DTL loss $L_{DTL}\left(\Delta ;\Sigma ,\Sigma^{*}\right)=\frac{1}{4}\left(\langle \Sigma \Delta ,\Delta \Sigma^{*}\rangle +\langle \Sigma^{*}\Delta ,\Delta \Sigma \rangle \right)+\langle \Delta ,\Sigma^{*}-\Sigma \rangle$, which is proposed by Yuan *et al*. [29] to estimate the differential network Δ when the absolute abundances are known. Again, our DCDTr loss takes the advantage of the bridge Eq (3) to avoid the unobserved absolute abundance and account for the compositionality. From another perspective, we can arrive at our DCDTr loss (10) by substituting the approximation Σ ≈ *G*Σ_ln ***x***_ *G*, Σ* ≈ *G*Σ_ln x*_ *G* into DTL loss. In the numerical experiments of section 3, we also investigated the performance of procedures which combine the approximation Σ ≈ *G*Σ_ln ***x***_ *G*, Σ* ≈ *G*Σ_ln x*_ *G* with other methods for differential network estimation, including the *ℓ*_1_-minimization method [28] for direct estimation of differential networks and joint graphical lasso (FGL, GGL) [27] for joint estimation of precision matrices. The detailed formulas are left in S1 Appendix.

Under the exchangeable condition *G*Σ = Σ*G* and *G*Σ* = Σ*G, it is easy to check that

$$\begin{array}{c} \begin{array}{cc} L_{DCDTr}\left(\Delta ;\Sigma ,\Sigma^{*}\right)= & \frac{1}{4}{\left\|{\left(G\Sigma G\right)}^{1/2}\left(\Delta -\left(\Sigma^{*-1}-\Sigma^{-1}\right)\right){\left(G\Sigma G\right)}^{*1/2}\right\|}_{F}^{2}+\\ & \frac{1}{4}{\left\|{\left(G\Sigma G\right)}^{*1/2}\left(\Delta -\left(\Sigma^{*-1}-\Sigma^{-1}\right)\right){\left(G\Sigma G\right)}^{1/2}\right\|}_{F}^{2}+\\ & \frac{1}{2}\left\langle G\left(\Sigma^{*}-\Sigma \right),\left(\Sigma^{*-1}-\Sigma^{-1}\right)G\right\rangle . \end{array} \end{array}$$ (11)

Obviously, Δ = Σ*^−1^ − Σ^−1^ is a minimizer of our DCDTr loss *L*_*DCDTr*_. In practical applications, we incorporate the finite sample estimators of Σ, Σ* and *ℓ*_1_ penalty into DCDTr loss, and our sparse estimator for the differential network Δ is proposed as

$$\begin{array}{c} \begin{array}{cc} & {\hat{\Delta }}_{\text{DCDTr}}=\underset{\Delta =\Delta^{T}}{\text{argmin}}\frac{1}{4}(\left\langle G{\hat{\Sigma }}_{\text{ln}\;x}G\Delta ,\Delta G{\hat{\Sigma }}_{\text{ln}\;x^{*}}G\right\rangle +\\ & \left\langle G{\hat{\Sigma }}_{\text{ln}\;x^{*}}G\Delta ,\Delta G{\hat{\Sigma }}_{\text{ln}\;x}G\right\rangle )+\left\langle \Delta ,G\left({\hat{\Sigma }}_{\text{ln}\;x^{*}}-{\hat{\Sigma }}_{\text{ln}\;x}\right)G\right\rangle +\lambda {\left|\Delta \right|}_{1}. \end{array} \end{array}$$ (12)

The tuning parameter λ is selected by minimizing the Bayesian Information Criterion (BIC) [28–30] as

$$\begin{array}{c} \begin{array}{cc} \text{BIC}= & \left(n+n^{*}\right)\|\frac{1}{2}\left(G{\hat{\Sigma }}_{\text{ln}\;x^{*}}G\Delta G{\hat{\Sigma }}_{\text{ln}\;x}G+G{\hat{\Sigma }}_{\text{ln}\;x}G\Delta G{\hat{\Sigma }}_{\text{ln}\;x^{*}}G\right)+\\ & G\left({\hat{\Sigma }}_{\text{ln}\;x^{*}}-{\hat{\Sigma }}_{\text{ln}\;x}\right){G\|}_{1}+\text{log}\left(n+n^{*}\right){\left|\Delta \right|}_{0}, \end{array} \end{array}$$ (13)

where |Δ|_0_ is the number of non-zero elements in the upper-triangle of Δ, and *n* and n* are the sample size.

Taking advantage of the algorithm developed by Yuan *et al*. [29] for the numerical solution of lasso penalized DTL loss estimator, the algorithm for the numerical solution of (12) is straightforward, essentially because we can simply replace $\hat{\Sigma }$ and ${\hat{\Sigma }}^{*}$ in DTL loss with $G{\hat{\Sigma }}_{\text{ln}\;x}G$ and $G{\hat{\Sigma }}_{\text{ln}\;x^{*}}G$ in our DCDTr loss. Following the idea of Yuan *et al*. [29], we introduce three new matrices Δ_1,2,3_ and Lagrangian multipliers Λ_1,2,3_, *ρ* for the solution of (12). The steps of the ADMM algorithm for the lasso penalized DCDTr loss estimator are presented as follows.

(a) Initialization: k = 0, $\Delta_{1}^{0},\Delta_{2}^{0},\Delta_{3}^{0},\Lambda_{1}^{0},\Lambda_{1}^{0}$ and $\Lambda_{3}^{0}$;
(b) $\Delta_{1}^{k+1}=K\left(G{\hat{\Sigma }}_{\text{ln}\;x}G,G{\hat{\Sigma }}_{\text{ln}\;x^{*}}G,2\rho \Delta_{3}^{k}+2\rho \Delta_{2}^{k}+G\left({\hat{\Sigma }}_{\text{ln}\;x}-{\hat{\Sigma }}_{\text{ln}\;x^{*}}\right)G+2\Lambda_{1}^{k}-2\Lambda_{3}^{k},4\rho \right)$;
(c) $\Delta_{2}^{k+1}=K\left(G{\hat{\Sigma }}_{\text{ln}\;x^{*}}G,G{\hat{\Sigma }}_{\text{ln}\;x}G,2\rho \Delta_{3}^{k}+2\rho \Delta_{1}^{k}+G\left({\hat{\Sigma }}_{\text{ln}\;x}-{\hat{\Sigma }}_{\text{ln}\;x^{*}}\right)G+2\Lambda_{3}^{k}-2\Lambda_{2}^{k},4\rho \right)$;
(d) $\Delta_{3}^{k+1}=S\left(\frac{1}{2\rho }\left(\rho \Delta_{1}^{k+1}+\rho \Delta_{2}^{k+1}-\Lambda_{1}^{k}+\Lambda_{2}^{k}\right),\frac{\lambda }{2\rho }\right)$;
(e) $\Lambda_{1}^{k+1}=\Lambda_{1}^{k}+\rho \left(\Delta_{3}^{k+1}-\Delta_{1}^{k+1}\right)$, $\Lambda_{2}^{k+1}=\Lambda_{2}^{k}+\rho \left(\Delta_{2}^{k+1}-\Delta_{3}^{k+1}\right)$ and $\Lambda_{3}^{k+1}=\Lambda_{3}^{k}+\rho \left(\Delta_{1}^{k+1}-\Delta_{2}^{k+1}\right)$;
(f) k = k+1;
(g) Repeat (b)-(f) until convergence.

The definitions of matrix operators *K*(*X*) and *S*(*X*) are listed in S1 Appendix.

## 3 Numerical results

In this section, we conduct several numerical experiments under different settings and compare them with other state-of-the-art methods. Given mean ***μ***_*p*_ and precision matrix Θ, we first generate the log-transformed absolute abundance ln ***z***_*i*_ = (ln *z*_*i*1_, ln *z*_*i*2_, …, ln *z*_*ip*_) with the multivariate normal distribution $N_{p}\left(\mu_{p},\Theta^{-1}\right)$, and then the relative abundances are $x_{i}=\left(\frac{z_{i1}}{\sum_{k=1}^{p}z_{ik}},\frac{z_{i2}}{\sum_{k=1}^{p}z_{ik}},\ldots ,\frac{z_{ip}}{\sum_{k=1}^{p}z_{ik}}\right)$, *i* = 1, 2, …, *n*. For another given mean $\mu_{p}^{*}$ and precision matrix Θ* under a new condition, the samples $x_{i}^{*}$, *i* = 1, 2, …, *n* are similarly generated. In the following simulations, we take *p* = 50 and ***μ***_*p*_ sampled from the uniform distribution $U_{p}\left(-0.5,0.5\right)$.

### 3.1 Simulations for CDTr loss

To investigate the performance of CDTr loss and the influence of the exchangeable condition, we considered the following network structures for Θ.

1. *Band graph*:
  $$\begin{array}{c} \theta_{ij}=\{\begin{array}{cc} 1, & |i-j|=1\;\text{or}\;p-1\\ -1, & |i-j|=2\;\text{or}\;p-2\\ 0, & \text{otherwise}. \end{array} \end{array}$$
2. *Cluster graph*: Divide *p* nodes into 5 clusters evenly. The nodes in different clusters are not connected, while the network for each cluster is the same as matrix *C* = (*c*_*ij*_)_10×10_, where
  $$\begin{array}{c} c_{ij}=\{\begin{array}{cc} 1, & 1\leq |i-j|\leq 5\\ -1, & 6\leq |i-j|\leq 10\\ 0, & \text{otherwise}. \end{array} \end{array}$$

The link strength is uniformly distributed in [*l*, *u*]. To be specific, *θ*_*ij*_ is replaced with *θ*_*ij*_*s*_*ij*_, where $s_{ij}∼U\left(l,u\right)$. We take (*l*, *u*) = (0.1, 0.1), (0.05, 0.15) and (0.0.2) separately to study the performance of CDTr loss when the exchangeable condition is satisfied by different degrees. These scenarios are named as Band-exact (Band-e), Band-approx1 (Band-a1), Band-approx2 (Band-a2) and Cluster-exact (Cluster-e), Cluster-approx1 (Cluster-a1), Cluster-approx2 (Cluster-a2), respectively. To obtain a positive definite precision matrix Θ, we first compute the smallest eigenvalue of Θ (denoted by *e*); then the diagonal elements of Θ are set as |*e*| + 0.3. The deviation to the exchangeable condition is measured with *dev* = ‖*G*Σ − Σ*G*‖_F_. The deviations under the aforementioned six scenarios are listed in Table 1. For each combination of the six network structures and four sample sizes *n* = 50, 100, 150, 200, a total of 100 datasets are generated and used to recover the network structure. Four state-of-the-art methods for network recovery are investigated, including gCoda [14], CD-trace [24], SPIEC(MB) and SPIEC(GL) [12]. We further consider an approximation method called aCDTr, which approximates Σ with *G*Σ_ln ***x***_ *G* [12] and employs D-trace loss to estimate Θ = Σ^−1^. Specifically, the estimator of aCDTr is

$$\begin{array}{c} {\hat{\Theta }}_{\text{aCDTr}}=\underset{\Theta ≻0,\Theta =\Theta^{T}}{\text{argmin}}\frac{1}{2}\left\langle \Theta^{2},G{\hat{\Sigma }}_{\text{ln}\;x}G\right\rangle -\left\langle \Theta ,I\right\rangle +\lambda {\left|\Theta \right|}_{1,\text{off}}. \end{array}$$ (14)

The true positive rate and true negative rate are evaluated at different tuning parameters and used to generate the receiver operating characteristic (ROC) curve. We use the area under the curve (AUC) to quantify the ability to recover the true underlying network.

**Table 1:** Deviations from the exchangeable condition under different scenarios.

Network	Band-e	Band-a1	Band-a2	Cluster-e	Cluster-a1	Cluster-a2
*dev*	0	0.203	0.348	0	0.109	0.205

In Table 2, we present the mean AUC scores of the above-mentioned methods under different settings. The mean AUC scores of CDTr and aCDTr are superior to the other four methods in all cases, even when the exchangeable condition does not hold exactly, which implies that CDTr and aCDTr outperform other methods in direct interaction network recovery. Moreover, the mean AUC of CDTr is slightly higher than that of aCDTr, except for the cluster graph and sample size *n* = 50. With increasing deviation, the performance of CDTr and aCDTr decreases, which is reasonable if the exchangeable condition does not exactly hold. Interestingly, the performance for the other four methods also decreases with increasing deviation. For all network structures and methods, the mean AUC scores increase as the sample size increases.

**Table 2:** The mean AUC scores of different methods under different settings.

n	Method	Network Structure					
		Band-e	Band-a1	Band-a2	Cluster-e	Cluster-a1	Cluster-a2
50	SPIEC(MB)	0.662	0.662	0.663	0.641	0.628	0.617
	SPIEC(GL)	0.694	0.695	0.690	0.696	0.677	0.660
	gCoda	0.689	0.688	0.683	0.679	0.660	0.645
	CD-trace	0.691	0.690	0.685	0.682	0.662	0.646
	aCDTr	0.727	0.729	0.717	**0.817**	**0.781**	**0.748**
	CDTr	**0.732**	**0.733**	**0.720**	0.816	0.780	0.746
100	SPIEC(MB)	0.765	0.760	0.742	0.722	0.705	0.688
	SPIEC(GL)	0.809	0.801	0.776	0.793	0.769	0.743
	gCoda	0.812	0.803	0.775	0.782	0.759	0.731
	CD-trace	0.813	0.802	0.774	0.782	0.758	0.730
	aCDTr	0.848	0.838	0.809	0.932	**0.902**	**0.859**
	CDTr	**0.857**	**0.846**	**0.816**	**0.933**	**0.902**	**0.859**
150	SPIEC(MB)	0.821	0.811	0.786	0.768	0.753	0.729
	SPIEC(GL)	0.870	0.857	0.822	0.845	0.823	0.791
	gCoda	0.882	0.864	0.825	0.841	0.816	0.783
	CD-trace	0.880	0.861	0.822	0.841	0.814	0.781
	aCDTr	0.908	0.894	0.855	0.972	0.950	0.910
	CDTr	**0.919**	**0.904**	**0.864**	**0.973**	**0.951**	**0.911**
200	SPIEC(MB)	0.858	0.848	0.814	0.799	0.781	0.757
	SPIEC(GL)	0.909	0.894	0.851	0.877	0.856	0.823
	gCoda	0.926	0.905	0.855	0.879	0.856	0.816
	CD-trace	0.922	0.902	0.851	0.873	0.849	0.806
	aCDTr	0.943	0.929	0.884	0.988	0.973	0.940
	CDTr	**0.955**	**0.940**	**0.893**	**0.989**	**0.975**	**0.942**

We further conducted several experiments on the following six representative network structures, without considering the exchangeable condition.

1. *Random graph*: Two nodes are connected with probability 0.1, and the strength is generated from a uniform distribution in [−0.2, −0.1] ∪ [0.1, 0.2].
2. *Band graph*: Connect pair (*i*, *j*) with strength uniformly distributed in [0.05*m* − 0.3, 0.05*m* − 0.25] ∪ [0.25 − 0.05*m*, 0.3 − 0.05*m*], if |*i* − *j*| = *m*, *m* = 1, 2, 3, 4.
3. *Neighbor graph*: Select *p* points from U(0,1) and connect the 5 nearest neighbors for each point with strength sampled from a uniform distribution in [−0.15, −0.05] ∪ [0.05, 0.15].
4. *Scale-free graph*: A scale-free graph is produced, following the B-A algorithm [32]. The initial graph has two connected nodes, and each new node is connected to only one node in the existing graph with the probability proportional to the degree of the each node in the existing graph. This results in *p* edges in the graph, and the strength between connected nodes is generated from a uniform distribution in [−0.2, −0.1] ∪ [0.1, 0.2].
5. *Hub graph*: Partition the nodes into 3 disjoint groups evenly and select a node as hub for each group. The hubs are connected with the non-hubs in the same group with strength uniformly distributed in [−0.2, −0.1] ∪ [0.1, 0.2].
6. *Block graph*: Divide *p* nodes into 5 blocks evenly. Connect pairs in the same block with probability 0.3 and pairs in different blocks with probability 0.1. The strength between connected nodes is uniformly distributed in [−0.2, −0.1] ∪ [0.2, 0.1].

Similarly, the diagonal elements of Θ are set as |*e*| + 0.3, where *e* is the smallest eigenvalue of Θ. The deviations from the exchangeable condition of these networks are listed in Table 3.

**Table 3:** Deviations from the exchangeable condition of six different network structures.

Network	Random	Hub	Neighbor	Block	Band	Scale-free
dev	0.722	0.932	0.937	0.61	0.949	0.449

We generated 100 datasets for each setting and used them to estimate the true precision matrix. The mean AUC scores of different methods under different settings are shown in Table 4. We can see that CDTr performs better than other methods in all cases, while the results of aCDTr is comparable to those of gCoda and CD-trace, and the results of SPIEC(MB) and SPIEC(GL) are worse than the others. Note that we did not consider the exchangeable condition when we set up the networks, implying that CDTr still works, even when the the exchangeable condition does not hold. Although the objective functions and performances of CDTr and aCDTr are similar as shown in Tables 2 and 4, they are derived from two quite different perspectives. aCDTr is based on the approximation Σ ≈ *G*Σ_ln ***x***_ *G* and assumes that the inverse of *G*Σ_ln ***x***_ *G* also approximates the inverse of Σ. However, as Fang *et al*. [14] stated, this approximation depends strongly on the condition number of the inverse covariance matrix. CDTr does not need aforementioned approximation and can guarantee that the inverse of Σ minimizes CDTr loss exactly under the exchangeable condition. The meaning of CDTr is that it avoids the use of approximation assumptions and provides a different perspective for precision matrix estimation.

**Table 4:** The mean AUC scores of different methods under different settings.

		Network Structure					
n	Method	Random	Band	Neighbor	Scale-free	Hub	Block
50	SPIEC(MB)	0.630	0.615	0.599	0.671	0.647	0.613
	SPIEC(GL)	0.652	0.637	0.616	0.697	0.690	0.635
	gCoda	0.652	0.636	0.615	0.700	0.745	0.633
	CD-trace	0.650	0.627	0.615	0.685	0.708	0.630
	aCDTr	0.677	0.664	0.636	0.728	0.748	0.660
	CDTr	**0.681**	**0.670**	**0.641**	**0.729**	**0.757**	**0.664**
100	SPIEC(MB)	0.728	0.687	0.674	0.785	0.767	0.693
	SPIEC(GL)	0.758	0.712	0.697	0.809	0.812	0.723
	gCoda	0.766	0.717	0.703	0.811	0.866	0.729
	CD-trace	0.765	0.713	0.706	0.797	0.839	0.725
	aCDTr	0.778	0.737	0.714	0.827	0.860	0.746
	CDTr	**0.786**	**0.745**	**0.726**	**0.831**	**0.872**	**0.754**
150	SPIEC(MB)	0.782	0.731	0.713	0.845	0.834	0.746
	SPIEC(GL)	0.816	0.758	0.742	0.870	0.877	0.783
	gCoda	0.831	0.770	0.756	0.874	0.925	0.796
	CD-trace	0.830	0.770	0.761	0.868	0.909	0.793
	aCDTr	0.832	0.779	0.759	0.883	0.914	0.802
	CDTr	**0.844**	**0.790**	**0.773**	**0.889**	**0.926**	**0.814**
200	SPIEC(MB)	0.820	0.761	0.745	0.884	0.880	0.780
	SPIEC(GL)	0.856	0.791	0.778	0.909	0.921	0.820
	gCoda	0.876	0.806	0.800	0.913	0.955	0.836
	CD-trace	0.873	0.810	0.808	0.912	0.952	0.842
	aCDTr	0.870	0.809	0.792	0.917	0.945	0.835
	CDTr	**0.883**	**0.821**	**0.811**	**0.923**	**0.955**	**0.849**

### 3.2 Simulations for DCDTr loss

We investigate the performance of DCDTr loss with some experiments in this section. The first precision matrix Θ is generated as follows:

1. *Random graph*: For Θ, two nodes are connected with probability 0.5, and the strength is generated from a uniform distribution in [−0.4, −0.2] ∪ [0.2, 0.4].
2. *Band graph*: Connect pair (*i*, *j*) with strength uniformly distributed in [0.05*m* − 0.3, 0.05*m* − 0.25] ∪ [0.25 − 0.05*m*, 0.3 − 0.05*m*], if |*i* − *j*| = *m*, *m* = 1, 2, 3, 4.
3. *Neighbor graph*: Select *p* points from U(0,1) and connect the 10 nearest neighbors for each point with strength sampled from a uniform distribution in [−0.4, −0.2] ∪ [0.2, 0.4].
4. *Scale-free graph*: The scale-free graph is generated with the B-A algorithm [32]. The strength between connected nodes is generated from a uniform distribution in [−0.4, −0.2] ∪ [0.2, 0.4].
5. *Hub graph*: Partition the nodes into 3 disjoint groups evenly and select a node as hub for each group. The hubs are connected with the non-hubs in the same group with strength uniformly distributed in [−0.4, −0.2] ∪ [0.2, 0.4].
6. *Block graph*: Divide *p* nodes into 5 blocks evenly. Connect pairs in the same block with probability 0.5 and pairs in different blocks with probability 0.3. The strength between connected nodes is uniformly distributed in [−0.4, −0.2] ∪ [0.4, 0.2].

Then 10% of the connected pairs in Θ will change to an unconnected state, while the same number of unconnected pairs in Θ will change to a connected state, such that we get another precision matrix Θ*. For scale-free and hub graph, the ratio of change is 40% based on the sparsity of the two graphs. The diagonal elements of Θ and Θ* are set as |*e*| + 0.3, where *e* is the smallest eigenvalue of Θ or Θ*, respectively. The deviations from the exchangeable condition of Θ and Θ* are listed in Table 5. Therefor, the differential matrix Δ is Θ* − Θ. The two precision matrices Θ and Θ* are used to generate data separately. The aforementioned four methods, including DCDTr, FGL, GGL and *ℓ*_1_-M, are used to estimate the true differential matrix Δ. Similarly, we evaluate the true positive rate and true negative rate at different tuning parameters and then compute the area under the ROC curve (AUC). We take the sample size *n* = 100, 200, 300, 400 and repeat this procedure 100 times.

**Table 5:** Deviations from the exchangeable condition of six different network structures.

Network	Random	Band	Neighbor	Scale-free	Hub	Block
*dev*	0.56	0.56	0.89	0.36	1.07	1.23
dev*	0.48	1.02	1.03	0.39	0.49	0.99

Table 6 presents the mean AUC scores of different methods under different settings. We see that no method is generally better than the others in all cases. DCDTr performs better than other methods in random graph, neighbor graph and block graph, while GGL achieves higher AUC in scale-free and hub graph. With the increase of sample size, the advantage of DCDTr becomes increasingly significant. Generally speaking, our proposed DCDTr performs well in different network estimations.

**Table 6:** The mean AUC scores of different methods under different settings.

		Network Structure					
n	Method	Random	Band	Neighbor	Scale-free	Hub	Block
100	*ℓ*_1_-M	0.588	0.735	0.673	0.771	0.799	0.610
	FGL	0.566	0.760	**0.680**	0.830	0.848	0.578
	GGL	0.545	**0.768**	0.679	**0.845**	**0.862**	0.556
	DCDTr	**0.596**	0.732	0.677	0.769	0.789	**0.619**
200	*ℓ*_1_-M	0.662	0.834	0.790	0.890	0.902	0.701
	FGL	0.616	0.837	0.763	0.923	0.922	0.636
	GGL	0.566	**0.840**	0.752	**0.930**	**0.930**	0.591
	DCDTr	**0.673**	0.831	**0.792**	0.890	0.899	**0.711**
300	*ℓ*_1_-M	0.712	**0.877**	0.851	0.937	0.943	0.765
	FGL	0.654	0.865	0.808	0.952	0.950	0.682
	GGL	0.585	0.864	0.782	**0.954**	**0.952**	0.616
	DCDTr	**0.721**	0.875	**0.855**	0.938	0.944	**0.774**
400	*ℓ*_1_-M	0.754	**0.911**	0.894	0.963	0.964	0.813
	FGL	0.683	0.890	0.838	**0.966**	0.963	0.718
	GGL	0.595	0.882	0.803	0.965	0.962	0.635
	DCDTr	**0.765**	**0.911**	**0.897**	0.963	**0.965**	**0.822**

## 4 Real data analysis

In this section, we illustrate our proposed method with an application to mouse skin microbiome data [33]. A total of 261 mice were divided into 3 groups: 78 non-immunized controls (Control), 119 immunized healthy individuals (Healthy) and 64 immunized epidermolysis bullosa acquisita individuals (EBA), according to the health conditions of skin immunizations. The OTUs appearing in less than 50% of the samples are filtered out, and the samples with a number of nonzero OTU counts less than 50% of the total selected OTUs are also removed. We finally arrived at a dataset with *p* = 77 OTUs and *n* = 232 samples (63 Control, 114 Healthy and 55 EBA). We use Bayesian-multiplicative replacement [34–36] to impute zero counts and normalize the data to compositional data.

Since the the underlying true direct interaction networks were not available and the accuracy of estimated networks was unobtainable, we evaluated the performance of the proposed methods with reproducibility as Fang *et al*. [14] and Kurtz *et al*. [12] suggusted. More specifically, we first constructed a reference network *est*_1_ (precision matrix or differential matrix) with all data for each group and method. We then selected half of the samples randomly to estimate the precision matrix or differential matrix (denoted by *est*_2_) again. The reproducibility was measured by the fraction of overlapping edges shared by *est*_1_ and *est*_2_ in the reference network *est*_1_.

For each group and each method of precision matrix estimation, the procedure stated above was repeated 20 times. The mean reproducibility is summarized in Table 7. CDTr and aCDTr outperformed the other four methods in terms of reproducibility in all three groups, implying that CDTr and aCDTr are more stable and accurate in direct interaction estimation. We also estimated the differential network for the Control-Healthy and Control-EBA groups, and the evaluation procedure was also repeated 20 times. The mean reproducibility is listed in Table 8. The highest reproducibility of DCDTr also implies that DCDTr is more stable and accurate in differential network estimation.

**Table 7:** The mean reproducibility for various methods and groups.

	SPIEC(MB)	SPIEC(GL)	gCoda	CD-trace	aCDTr	CDTr
Control	0.47	0.55	0.57	0.58	0.59	0.62
Healthy	0.55	0.62	0.59	0.80	0.83	0.84
EBA	0.47	0.60	0.55	0.74	0.97	0.96

**Table 8:** The mean reproducibility for various methods and groups.

	*ℓ*_1_-M	FGL	GGL	DCDTr
Control-Healthy	0.72	0.53	0.54	0.78
Control-EBA	0.84	0.63	0.64	0.87

**Fig 1:**
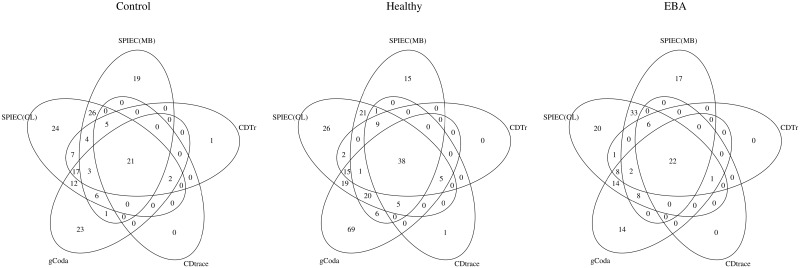
Venn diagrams of shared edges among direct interaction networks from various methods.

**Fig 2:**
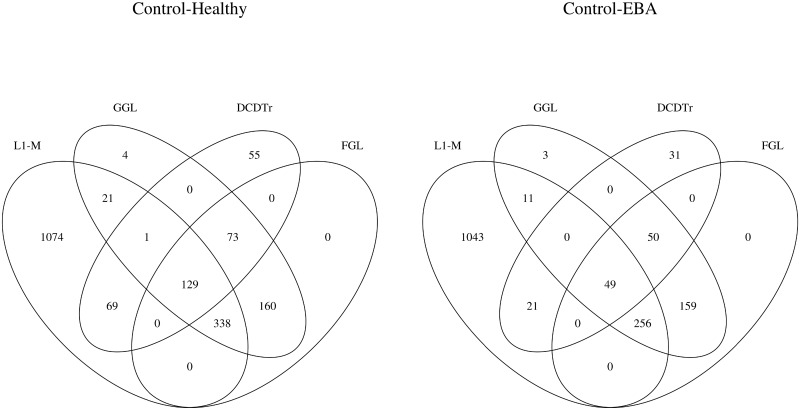
Venn diagrams of shared edges among differential networks from various methods.

Finally, we employed all methods to build a candidate microbiome association network from the unified dataset for each group and group pairs. In Fig 1, we present the number of shared edges for direct interaction networks recovered from various methods via Venn diagrams. We can see that the direct interaction network from CDTr is close to that of CD-trace, while the network from SPIEC(GL) and SPIEC(MB) are more similar. A total of 21, 38 and 22 edges are shared by all candidate networks for control, healthy and EBA groups, respectively, comprising the core interaction network among OTUs. Moreover, almost all direct interactions discovered by CDTr are in this core interaction network, while SPIEC(GL), SPIEC(MB) and gCoda discover some eccentric interactions. The number of shared edges for differential networks are shown in Fig 2. The situation for differential networks is much more complicated. *ℓ*_1_-M discovered many eccentric differential edges in both groups, but these were not confirmed by other methods. The differential edges from GGL and FGL are almost the same for both groups, and are more than the edges from DCDTr. Most differential edges from DCDTr were verified by both GGL and FGL for both groups, implying that DCDTr is good at inferring the crucial differential edges without mixing nonessential edges.

To investigate the influence of zeros in the compostional data, we first divide 77 variables into 7 sets evenly according to the proportion of nonzero measurements in each variable, and then calculate the percentage of nonzero measurements (named nonzero density) in each set. The average degree of variables (i.e., nodes) in the same set is computed with each network constructed by above-mentioned methods. The nonzero density and average degree for each set are summarized in Tables 9 and 10 for Control, Healthy, EBA and Control-Healthy, Control-EBA group, respectively. For Control and EBA group, the average degree tends to be bigger with larger nonzero density for all methods. When the nonzero density is 20% in Set1 for Control group and 49% in Set1 for EBA group, aCDTr and CDTr do not recover any connections with these rare abundance bacteria, which implies that the recovered connections are not due to zero corrections. For Healthy, Control-Healthy and Control-EBA group with fewer zeros in the data, the average degree does not show clear pattern and is more close to random distribution, which implies that zero measurements do not influence network inference significantly when zeros in compositional data are relatively few.

**Table 9:** The nonzero density and average degree for each set and networks constructed by various methods in control, healthy and EBA group.

Control							
	Set1	Set2	Set3	Set4	Set5	Set6	Set7
Nonzero Density	20%	45%	56%	67%	79%	90%	99%
SPIEC(MB)	0.55	1.46	1.40	1.75	2.50	2.90	4.54
SPIEC(GL)	0.00	1.27	1.80	1.58	4.80	5.20	7.92
gCoda	0.18	1.67	1.20	0.92	4.80	3.00	3.78
CD-trace	0.00	0.09	0.30	0.33	1.40	0.80	2.00
aCDTr	0.00	0.27	0.60	0.42	3.40	2.50	3.62
CDTr	0.00	0.36	0.60	0.50	3.20	2.50	3.62
Healthy							
	Set1	Set2	Set3	Set4	Set5	Set6	Set7
Nonzero Density	62%	69%	78%	83%	88%	95%	99%
SPIEC(MB)	2.54	1.44	3.92	3.00	2.91	2.30	4.33
SPIEC(GL)	5.09	1.56	4.77	2.64	4.00	3.10	7.33
gCoda	7.27	2.78	6.85	4.64	2.82	2.50	4.58
CD-trace	2.00	0.33	1.85	1.09	1.09	0.90	3.00
aCDTr	2.00	0.22	1.77	0.91	1.09	0.90	3.50
CDTr	2.64	0.33	2.08	1.00	1.36	0.90	3.83
EBA							
	Set1	Set2	Set3	Set4	Set5	Set6	Set7
Nonzero Density	49%	60%	66%	73%	83%	89%	99%
SPIEC(MB)	0.78	2.30	1.56	1.81	2.00	3.10	4.17
SPIEC(GL)	0.67	3.10	2.33	2.12	2.46	4.40	5.58
gCoda	0.89	2.20	1.44	1.44	1.18	3.00	2.42
CD-trace	0.00	0.80	0.11	0.38	0.36	2.00	1.58
aCDTr	0.00	1.20	0.11	0.56	0.73	2.60	2.00
CDTr	0.00	1.20	0.11	0.56	0.73	2.60	2.00

**Table 10:** The nonzero density and average degree for each set and networks constructed by various methods in Control-Healthy and Control-EBA group.

Control-Healthy							
	Set1	Set2	Set3	Set4	Set5	Set6	Set7
Non-zero Density	58%	64%	67%	74%	82%	91%	99%
*ℓ*_1_-M	35.82	39.45	44.40	50.91	47.25	40.82	37.82
FGL	18.27	18.09	18.80	21.27	20.33	15.82	14.54
GGL	18.55	18.64	19.80	22.00	20.75	16.55	15.64
DCDTr	10.54	7.91	10.00	11.46	9.00	7.36	3.27
Control-EBA							
	Set1	Set2	Set3	Set4	Set5	Set6	Set7
Non-zero Density	44%	52%	61%	69%	77%	87%	98%
*ℓ*_1_-M	30.50	26.00	36.09	43.73	37.70	38.64	38.58
FGL	10.80	11.17	15.00	16.82	14.00	13.54	12.25
GGL	10.90	11.25	15.18	17.45	14.10	14.18	13.00
DCDTr	4.10	4.33	6.73	5.09	4.10	2.09	1.25

## 5 Conclusion

Inferring the direct interactions among microbial species and understanding how the network structure changes are important in the study of ecology and medicine. In this paper, we propose two loss functions to estimate the direct interaction network and differential network from compositional microbial data based on clr transformation and D-trace loss for absolute abundance data. Although the proposed CDTr loss and DCDTr loss are derived from an exchangeable condition, we show that they still perform well and better than other methods under different scenarios in our numerical simulations. However, the reasonableness of the exchangeable condition should be further examined in theory and biology. Finally, the consistency of the estimators does not come with a theoretical guarantee, which is a common limitation of gCoda, SPIEC, CDTr and DCDTr. For future work, we are interested in developing theorems about the consistency property in both direct interaction network and differential network estimation.

## Supporting information

S1 Appendix Supplementary for compositional data analysis via lasso penalized D-trace loss. The matrix operators *S*(*X*),*K*(*X*),*H*(*X*) and [*X*]_+_ used in Algorithm 1 and Algorithm 2 for the numerical solutions of lasso penalized CDTr and DCDTr loss are presented in this Supplementary. We also demonstrate the relationship between D-trace loss and CDTr loss, as well as the relationship between DTL loss and DCDTr loss. The detailed formulas of *ℓ*_1_-minimization method and joint graphical lasso (FGL, GGL) are listed in this Supplementary.
(PDF)
