# Supplementary material for: Direct interaction network and differential network inference from compositional data via lasso penalized D-trace loss
Source: PLoS One. 2019 Jul 24;14(7):e0207731. doi: 10.1371/journal.pone.0207731 (PMC6655598; doi:10.1371/journal.pone.0207731)
Supplement: S1 Appendix — The matrix operators S(X),K(X),H(X) and [X]+ used in Algorithm 1 and Algorithm 2 for the numerical solutions of lasso penalized CDTr and DCDTr loss are presented in this Supplementary. We also demonstrate the relationship between D-trace loss and CDTr loss, as well as the relationship between DTL loss and DCDTr loss. The detailed formulas of ℓ1-minimization method and joint graphical lasso (FGL, GGL) are listed in this Supplementary. (PDF) [file pone.0207731.s001.pdf]

Supplements for Compositional Data Analysis via Lasso Penalized D-trace Loss

Shun He[1], Minghua Deng[1,2*],

**1** School of Mathematical Sciences, Peking University, Beijing, 10087, P.R.China
**2** Center for Statistical Science, Peking University, Beijing, 10087, P.R.China

* dengmh@math.pku.edu.cn

# 1 Matrix Operators for Numerical Solution Algorithms

We present some matrix operators for convenience, which are used in Algorithm 1 and Algorithm 2 for the numerical solutions of lasso penalized CDTr and DCDTr loss. Given a symmetric matrix $A$ with eigenvalue decompositions $A = U_A \text{diag}(\sigma_1, \dots, \sigma_p) U_A^T$, let $H(A, B) = U_A\{(U_A^T B U_A) \circ C\} U_A^T$, where $C_{ij} = 2/(\sigma_i + \sigma_j)$ and $\circ$ denotes the Hadamard product of two matrices. We difine the matrix operator $[A]_+$ as $[A]_+ = U_A \text{diag}\{\max(\sigma_1, \epsilon), \dots, \max(\sigma_p, \epsilon)\} U_A^T$. For a matrix $X$, the shrinkage operator $S$ is defined by

$$S(X, \lambda)_{i,j} = \begin{cases} X_{i,j} - \lambda, & X_{i,j} > \lambda, \\ X_{i,j} + \lambda, & X_{i,j} < -\lambda, \\ 0, & -\lambda \le X_{i,j} \le \lambda. \end{cases}$$

Let $A = U_A \Sigma_A U_A^T$ and $B = U_B \Sigma_B U_B^T$ be the eigenvalue decompositions of the symmetric matrices $A$ and $B$, respectively. We define $K(A, B, C, \gamma) = U_A[D \circ (U_A^T C U_B^T)] U_B$, where $D_{ij} = (\sigma_j^A \sigma_i^B + \gamma)^{-1}$ and $\circ$ denotes the Hadamard product of two matrices.

# 2 CDTr loss and D-trace loss

The original D-trace loss proposed by Zhang and Zhou [4] is

$$\begin{aligned} L_D(\Theta; \Sigma) &= \frac{1}{2}\langle \Theta^2, \Sigma \rangle - \langle \Theta, I \rangle \\ &= \frac{1}{2}\|\Sigma^{1/2}\Theta - \Sigma^{-1/2}\|_{\text{F}}^2 - \frac{1}{2}\langle \Sigma^{-1}, I \rangle, \end{aligned} \tag{1}$$

which is used to estimate the precision matrix from absolute abundance data. The first term in (1) is 0 if and only if $\Sigma^{1/2}\Theta - \Sigma^{-1/2} = 0$, i.e., $\Theta = \Sigma^{-1}$. For compositional data, we consider $\Theta G = \Sigma^{-1} G$ to account for the compositionality. Under the exchangeable condition $G\Theta = \Theta G$, it implies $\Sigma^{1/2} G \Theta - \Sigma^{-1/2} G = 0$. So, our CDTr loss is constructed as

$$\begin{aligned} L_{CD}(\Theta; \Sigma) &= \frac{1}{2}\|\Sigma^{1/2} G \Theta - \Sigma^{-1/2} G\|_{\text{F}}^2 - \frac{1}{2}\langle \Sigma^{-1}, G \rangle \\ &= \frac{1}{2}\langle \Theta^2, G \Sigma G \rangle - \langle \Theta, G \rangle. \end{aligned} \tag{2}$$

It is obviously that D-trace loss and CDTr loss have similar structure and meaning. Moreover, the term $G\Sigma G$ in CDTr is the same as $G\Sigma_{\ln \boldsymbol{x}}G$ for compositional data, which makes it easy to be estimated with compositional samples, even when the absolute abundances are not available. Note that CDTr loss does not mean approximating $\Sigma$ with $G\Sigma_{\ln \boldsymbol{x}}G$ [2] and using D-trace loss to estimate $\Theta = \Sigma^{-1}$, which yields

$$L_{aCDTr} = \frac{1}{2}\langle \Theta^2, G\Sigma G\rangle - \langle \Theta, I\rangle. \tag{3}$$

# 3 DCDTr loss and DTL loss

The original DTL loss proposed by Yuan *et al.* [3] is

$$\begin{aligned} L_{DTL}(\Delta;\Sigma,\Sigma^*) =& \frac{1}{4}(\langle \Sigma\Delta, \Delta\Sigma^*\rangle + \langle \Sigma^*\Delta, \Delta\Sigma\rangle) + \langle \Delta, \Sigma^* - \Sigma\rangle \\ =& \frac{1}{4}\|\Sigma^{1/2}(\Delta - (\Sigma^{*-1} - \Sigma^{-1}))\Sigma^{*1/2}\|_{\mathrm{F}}^2 + \\ & \frac{1}{4}\|\Sigma^{*1/2}(\Delta - (\Sigma^{*-1} - \Sigma^{-1}))\Sigma^{1/2}\|_{\mathrm{F}}^2 + \\ & \frac{1}{2}\langle \Sigma^* - \Sigma, \Sigma^{*-1} - \Sigma^{-1}\rangle, \end{aligned} \tag{4}$$

which is used to estimate the differential network with absolute abundance data. From the first two terms, it is easy to see that DTL loss is minimized if and only if $\Sigma^{1/2}(\Delta - (\Sigma^{*-1} - \Sigma^{-1}))\Sigma^{*1/2} = 0$ and $\Sigma^{*1/2}(\Delta - (\Sigma^{*-1} - \Sigma^{-1}))\Sigma^{1/2} = 0$, which implies $\Delta - (\Sigma^{*-1} - \Sigma^{-1}) = 0$. Similarly, we construct the equation $G\Sigma^{1/2}(\Delta - (\Sigma^{*-1} - \Sigma^{-1}))\Sigma^{*1/2}G = 0$ and $G\Sigma^{*1/2}(\Delta - (\Sigma^{*-1} - \Sigma^{-1}))\Sigma^{1/2}G = 0$ for compositional data. Under the exchangeable condition $G\Sigma = \Sigma G$ and $G\Sigma^* = \Sigma^* G$ and using $G^2 = G$, we can get $G\Sigma^{1/2} = \Sigma^{1/2}G = (G\Sigma G)^{1/2}$ and $G\Sigma^{*1/2} = \Sigma^{*1/2}G = (G\Sigma G)^{*1/2}$. Thus, our DCDTr loss is proposed as

$$\begin{aligned} & L_{DCDTr}(\Delta;\Sigma,\Sigma^*) = \\ & \frac{1}{4}\|(G\Sigma G)^{1/2}(\Delta - (\Sigma^{*-1} - \Sigma^{-1}))(G\Sigma G)^{*1/2}\|_{\mathrm{F}}^2 + \\ & \frac{1}{4}\|(G\Sigma G)^{*1/2}(\Delta - (\Sigma^{*-1} - \Sigma^{-1}))(G\Sigma G)^{1/2}\|_{\mathrm{F}}^2 + \\ & \frac{1}{2}\langle G(\Sigma^* - \Sigma), (\Sigma^{*-1} - \Sigma^{-1})G\rangle = \\ & \frac{1}{4}(\langle G\Sigma G\Delta, \Delta G\Sigma^* G\rangle + \langle G\Sigma^* G\Delta, \Delta G\Sigma G\rangle) + \langle \Delta, G(\Sigma^* - \Sigma)G\rangle. \end{aligned} \tag{5}$$

Similarly, according to $G\Sigma G = G\Sigma_{\ln \boldsymbol{x}}G$ and $G\Sigma^* G = G\Sigma_{\ln \boldsymbol{x}^*}G$, the terms $G\Sigma G$ and $G\Sigma^* G$ can be estimated with compositional samples, even when the absolute abundances are not available.

# 4 Other Methods For Differential Network Estimation

The $\ell_1$-minimization method [5] and joint graphical lasso (FGL, GGL) [1] are two methods for differential network estimation when the absolute abundances are available. The original optimization problem of $\ell_1$-minimization method is

$$\hat{\Delta}_{\ell_1\text{-M}} = \operatorname{argmin} \|\Delta\|_1 \quad \text{s.t.} \quad \|\hat{\Sigma}^* \otimes \hat{\Sigma} vec(\Delta) - vec(\hat{\Sigma}^* - \hat{\Sigma})\|_\infty \leq \lambda,$$

where $\lambda_1$ and $\lambda_2$ are tuning parameters. We use the approximations $\Sigma \approx G\Sigma_{\ln \boldsymbol{x}}G, \Sigma^* \approx G\Sigma_{\ln \boldsymbol{x}^*}G$, which yields the following optimization problem for differential network estimation with compositional data,

$$\begin{aligned}&\hat{\Delta}_{\ell_1\text{-M}} = \operatorname{argmin} |\Delta|_1 \quad \text{s.t.}\\ &\|(G\hat{\Sigma}_{\ln \boldsymbol{x}^*}G) \otimes (G\hat{\Sigma}_{\ln \boldsymbol{x}}G)vec(\Delta) - vec(G(\hat{\Sigma}_{\ln \boldsymbol{x}^*} - \hat{\Sigma}_{\ln \boldsymbol{x}})G)\|_\infty \leq \lambda,\end{aligned}$$

Similarly, the original objective function of joint graphical lasso (FGL, GGL) is

$$L_{JGL}(\Theta, \Theta^*) = n[\log(\det \Theta) - tr(\Sigma\Theta)] + n^*[\log(\det \Theta^*) - tr(\Sigma\Theta^*)].$$

With the approximations above, the objective function for differential network estimation with compositional data turns into

$$\begin{aligned}L_{JGL}(\Theta, \Theta^*) = n[\log(\det \Theta) - tr(G\Sigma_{\ln \boldsymbol{x}}G\Theta)] + \\ n^*[\log(\det \Theta^*) - tr(G\Sigma_{\ln \boldsymbol{x}^*}G\Theta^*)].\end{aligned}$$

The corresponding FGL estimator and GGL estimator are

$$\begin{aligned}[\hat{\Theta}_{\text{FGL}}, \hat{\Theta}^*_{\text{FGL}}] = &\underset{\Theta \succ 0, \Theta = \Theta^{\text{T}}, \Theta^* \succ 0, \Theta = \Theta^{*\text{T}}}{\operatorname{argmin}} \{L_{JGL}(\Theta, \Theta^*) + \\ &\lambda_1 \sum_{i \neq j} (\theta_{ij} + \theta^*_{ij}) + \lambda_2 \sum_{i,j} |\theta_{ij} - \theta^*_{ij}|\}\\ \hat{\Delta}_{\text{FGL}} = &\hat{\Theta}^*_{\text{FGL}} - \hat{\Theta}_{\text{FGL}}\\ [\hat{\Theta}_{\text{GGL}}, \hat{\Theta}^*_{\text{GGL}}] = &\underset{\Theta \succ 0, \Theta = \Theta^{\text{T}}, \Theta^* \succ 0, \Theta = \Theta^{*\text{T}}}{\operatorname{argmin}} \{L_{JGL}(\Theta, \Theta^*) + \\ &\lambda_1 \sum_{i \neq j} (\theta_{ij} + \theta^*_{ij}) + \lambda_2 \sum_{i \neq j} (\theta^2_{ij} + \theta^{*2}_{ij})^{1/2}\}\\ \hat{\Delta}_{\text{GGL}} = &\hat{\Theta}^*_{\text{GGL}} - \hat{\Theta}_{\text{GGL}},\end{aligned} \tag{6}$$

where $\lambda_1$ and $\lambda_2$ are tuning parameters.

# References


1. Patrick Danaher, Pei Wang, and Daniela M. Witten. The joint graphical lasso for inverse covariance estimation across multiple classes. *Journal of the Royal Statistical Society: Series B (Statistical Methodology)*, 76(2):373–397, 2014.

2. Zachary D Kurtz, Christian L Müller, Emily R Miraldi, Dan R Littman, Martin J Blaser, and Richard A Bonneau. Sparse and compositionally robust inference of microbial ecological networks. *PLoS computational biology*, 11(5):e1004226, 2015.

3. Huili Yuan, Ruibin Xi, and Minghua Deng. Differential network analysis via the lasso penalized d-trace loss. *Biometrika*, 104(4), 2015.

4. Teng Zhang and Hui Zou. Sparse precision matrix estimation via lasso penalized d-trace loss. *Biometrika*, 101(1):103–120, 2014.

5. Sihai Dave Zhao, T. Tony Cai, and Hongzhe Li. Direct estimation of differential networks. *Biometrika*, 101(2):253–268, 2014.
